# “*It's Just Presence,”* the Contributions of Aboriginal and Torres Strait Islander Health Professionals in Cancer Care in Queensland

**DOI:** 10.3389/fpubh.2018.00344

**Published:** 2018-12-18

**Authors:** Audra de Witt, Frances C. Cunningham, Ross Bailie, Nikki Percival, Jon Adams, Patricia C. Valery

**Affiliations:** ^1^Menzies School of Health Research, Brisbane, QLD, Australia; ^2^Division of Epidemiology and Health Systems, Centre for Primary Health Care Systems, Charles Darwin University, Darwin, NT, Australia; ^3^Population Health, QIMR Berghofer Medical Research Institute, Brisbane, QLD, Australia; ^4^University Centre for Rural Health, University of Sydney, Lismore, NSW, Australia; ^5^Faculty of Health, University of Technology Sydney, Sydney, NSW, Australia

**Keywords:** Aboriginal and Torres Strait Islanders, health professionals, culturally competent care, follow-up cancer care, qualitative methods

## Abstract

**Objectives:** The aim of this research was to explore health professionals' perspectives on the provision of follow-up cancer care for Aboriginal and Torres Strait Islander patients in Queensland.

**Methods:** Semi-structured interviews were conducted with Indigenous and non-Indigenous health professionals who had experience providing care for Indigenous cancer patients in the primary health care and hospital setting.

**Results:** Participants were recruited from six Aboriginal Community Controlled Health Services (*n* = 17) and from a tertiary hospital (*n* = 9) across urban, regional, and remote geographical settings. Culturally safe care, psychological support, determining patient needs, practical assistance, and advocating for Indigenous health were identified as enablers to support the needs of Indigenous patients when accessing cancer care, and Indigenous health professionals were identified as the key enabler.

**Conclusion:** Indigenous health professionals significantly contribute to the provision of culturally competent follow-up cancer care by increasing the accessibility of follow-up cancer care services and by supporting the needs of Indigenous cancer patients. All health professionals need to work together and be sufficiently skilled in the delivery of culturally competent care to improve the Indigenous cancer journey and outcomes for Indigenous people. Effective organizational policies and practices are crucial to enable all health professionals to provide culturally competent and responsive cancer care to Indigenous Australians.

While overall cancer survival has improved in Australia over time, significant disparities in cancer outcomes exist between different population groups. Aboriginal and Torres Strait Islander people (hereafter, referred to as Indigenous Australians), have poorer cancer outcomes compared to non-Indigenous Australians. They experience disparities throughout the cancer continuum such as in cancer screening rates, (1) stage of diagnosis (1), and lower treatment and survival rates (2).

Many factors including higher smoking rates, poor nutrition, physical inactivity, and social disadvantage (such as lower employment rates and less education) (3) contribute toward the health gap between Indigenous and non-Indigenous Australians. Indigenous Australians are less likely to participate in population screening programs (such as for breast, cervical, and bowel cancer) and continue to experience difficulties accessing health services (3).

A recent review of risk factors for cancer in the Indigenous population found that people living in geographically remote areas and young Indigenous people (as opposed to elderly) were at increased risk of some cancers (such as esophageal and lung cancer) (4). However, there are variations in cancer definitions, sources of notifications and accuracy of records across state and territory cancer registries in Australia, and accurate measures of risk and cancer outcomes are limited for this population (4). There is also limited available information on the follow-up of Indigenous people with cancer upon discharge from hospital and their use of cancer related health services in the management of their care.

Significant variations in beliefs and perceptions regarding health, illness, well-being and death between Indigenous and non-Indigenous Australians influence health outcomes (5). Racism, distrust of health service providers, and communication challenges also influence health behaviors (6). Such issues can impact decisions to access health services, therefore having an understanding of such factors is key to providing more culturally competent care for Indigenous Australians (7).

A related study investigating Indigenous patients' perspectives on follow-up cancer care found that timely and quality knowledge exchange (such as discharge information), effective communication, and continuity and coordination of care between tertiary and primary health care (PHC) services were determined as crucial by Indigenous cancer patients, as was strong relationships between patients and health care providers across settings (8). In another study, hospital-based health professionals caring for Indigenous patients identified several enablers to the provision of cancer care. This included building trust and rapport with patients, ability to access an Indigenous liaison officer or Aboriginal health worker, maintaining relationships with Indigenous communities, availability of patient social supports, and helping patients navigate the health system (9). Indigenous patients with strong social supports across the cancer trajectory appeared to be more engaged in their cancer treatment and follow-up care, and were more likely to complete cancer treatment (9).

This paper aims to extend the existing literature through reporting on PHC health professionals' and tertiary-based health professionals' perspectives on the provision of follow-up cancer care in relation to accessing health services and meeting the needs of Indigenous patients. The overarching aim of the research study was to investigate the patterns of care for Indigenous patients diagnosed with cancer at the PHC setting and the systems in place to support high quality care within and across the primary, secondary and tertiary sector within the Queensland health service context. Several common terms are utilized in this paper, and to provide clarity, definitions of these terms in the context of this paper are provided. “Indigenous health professionals /staff” refers to all employees working at the PHC and hospital setting who identify as Indigenous. Cultural competency refers to the requirements of organizations in which “organizations have a defined set of values and principles, and demonstrate behaviors, attitudes, policies, and structures that enable them to work effectively cross-culturally” (10). Culturally responsive care in contrast, refers to the provision of patient-centered care by health service staff, which takes into account an individual's social and cultural background in recognition of its influence on the patient-provider interaction and care outcome (11).

## Materials and Methods

### Setting and Sample

Semi-structured interviews were conducted with Indigenous and non-Indigenous health professionals who had experience providing care for Indigenous patients diagnosed with cancer. PHC participants were recruited across urban, regional, and remote geographical locations and hospital participants were recruited from an urban setting. The study utilized a purposive sampling approach (12). PHC services recruited and referred to in this paper also participated in the larger cross-sectional patterns of cancer care study of Indigenous patients in Queensland. This overall study included the health professionals' interview study reported in this manuscript. The Indigenous patients' perspectives study on cancer follow-up care (8) and an audit study of patients' medical chart reviews (13) are described at length elsewhere. Health professionals who worked with cancer patients in a tertiary hospital in Brisbane were also invited to participate in this study.

### Data Collection and Procedure

Participants were interviewed at a time and place suitable to them between May 2015 and August 2016. A semi-structured interview format, the most frequently used technique for interviews in qualitative research (14), and in the health context (15) was utilized in this study. The interview guide was developed, piloted, and refined (based on pilot findings), and was used to cover key themes that were identified through review of the literature. The use of the semi-structured interview format enabled the interviewer to be more responsive to participants, and allowed for the investigation of relevant issues as they emerged (16). All interviews were audio-recorded and conducted face-to-face except for one, which was conducted over the telephone. The interview duration for PHC and tertiary health professional participants averaged 53 and 58 min, respectively, with a total of 1,370 interview minutes recorded for all study participants combined. Data collection ceased when data saturation was achieved (17) and as agreed upon by ADW and FC. All participants were provided with the opportunity to review their interview transcript. Open-ended questions were asked to determine health professionals' perspectives about the follow-up of cancer care for Indigenous patients at the PHC service and at the hospital. This included looking at the interface between the tertiary and PHC sectors. Interview topics explored included Indigenous cancer patients' needs, referrals to health services within and between the primary and tertiary sectors, and access to care in relation to patients' follow-up cancer care. The interviewer (ADW) was unknown to participants prior to the interview.

### Data Analysis

Interview recordings were de-identified, professionally transcribed and imported into NVivo (version 10). NVivo software was used to manage the data and to assist in the process of analysis. Thematic and inductive analysis was conducted. This involved an iterative process of data coding, reviewing an initial list of codes, organizing codes within themes, revising themes against codes for consistency and completeness until key themes were identified (18). A three step process for coding semi-structured interviews were utilized to ensure a high level of intercoder reliability (19). The first stage involved two researchers independently analyzing a sample of transcripts that were manually coded. These constructed codes were then compared for inconsistencies and for intercoder reliability (20). The second stage involved discussing coding disagreements to ensure a high level of intercoder agreement. In this study, there were no coding disagreements and a high level of intercoder agreement was established (ADW, AL, FC). The third stage involved utilizing the established and agreed codes on the full set of transcripts. A high level of inter-coder reliability, along with inclusion of representative quotes, contributed to research credibility and analytic rigor (19, 20). The consolidated criteria for reporting qualitative research (COREQ), a checklist to report on important aspects of a qualitative study, was used to guide the reporting of our findings (21).

### Ethics Approval

This research was approved by the Human Research Ethics Committees of Darling Downs Hospital and Health Service (HREC/14/QTDD/32), Menzies School of Research (HREC/2014-2222), QIMR Berghofer Medical Research Institute (HREC/P2127) and relevant Aboriginal community boards of participating services.

## Results

Seventeen PHC services were invited to contribute to the study by participating in health professional interviews. Of these, six Aboriginal Community Controlled Health Services (ACCHSs) agreed to participate and eleven services declined. (An ACCHS is a PHC service that delivers holistic, comprehensive and culturally appropriate health care to the community and is led by the local Indigenous community through a locally elected board of management) (22). Of those PHC centers that declined, two PHC centers were Queensland Health operated and nine were ACCHSs. We approached one tertiary hospital located in an urban setting that agreed to participate in this study. Health professionals were recruited from six ACCHSs across Queensland (*n* = 17) and from a tertiary hospital in Brisbane (*n* = 9) (see Table 1). In total, eleven health professionals interviewed identified as Indigenous (see Table 2).

**Table 1:** Participant characteristics.

	**Setting**	
	**PHC**	**Tertiary**
***SEX***		
Male	6	3
Female	11	6
***PROFESSION***		
Receptionist/admin officer	1	
Aboriginal health Workers/professionals	2	
Aboriginal liaison officer		2
Allied health coordinator	1	
Maternal and child health manager	1	
Enrolled nurse	2	
Registered nurse	4	1
Nutritionist		1
Social worker		1
General practitioner	6	
Medical oncologist		1
Radiation oncologist		2
Hematologist		1
***GEOGRAPHICAL LOCATION***		
Major city	1	1
Inner regional	1	
Outer regional	3	
Remote area	1	

**Table 2:** Indigenous staff members interviewed.

**Roles of Interviewed Health staff members who Identified as Indigenous**	
***PRIMARY HEALTH CARE SETTING:***	
Registered nurses	3
Enrolled nurse	1
Allied health coordinator	1
Receptionist/Administrative worker	1
Maternal and child health manager	1
Aboriginal health workers	2
***HOSPITAL SETTING:***	
Aboriginal liaison officers	2

Analysis of interviews identified several major themes as enablers for supporting the needs of Indigenous cancer patients in follow-up cancer care; culturally responsive care; psychological support (for patient and family); determining patient needs; provision of practical assistance (transport, accommodation and flexibility with hospital appointments) and advocating for Indigenous health. Indigenous health professionals were identified as being a key enabler across these five themes in the provision of cancer-related follow-up care at the PHC setting and at the interface between the PHC and hospital setting. This paper investigates the role of Indigenous health professionals in relation to these identified themes as described below. Exemplar quotes are used throughout the text. Additional supporting quotes are provided in Table 3.

**Table 3:** Sample participants' quotes in relation to theme.

**Theme**	**Representative respondent comments**
Culturally safe care	“*There was another lady who had this lump…she had it for about four or five months, She didn't say anything because she was waiting for me to do her health check*”. PHC Aboriginal Health worker (AHW) 17 “*If they've got a letter from the specialist, then usually we'll get hold of the patient … and bring 'em in to explain that they need to go…because sometimes they don't understand…it's the way that they're told”*. PHC Indigenous Enrolled Nurse (EN) 8 “*I worked in a non-Indigenous practice before coming here, and we here we have a lot more time per patient. If we were forced to work like a normal practice then it would be an absolute disaster.”* Non-Indigenous GP 11 “*it's all about being accessible and making the place culturally safe … a welcoming environment as patients are coming in”*. Hospital Aboriginal Liaison Officer (ALO) 7
Psychological support	***“**Yeah, I was with him every step of the way … he started his chemo and he was really frightened… I sat with him when he went through all of the different ways that he felt. That's the most important thing. If they've got someone there to support them then it's so much easier for them.”* PHC Indigenous Health worker 17 “*I looked after a young fellow who was in his late 30s … he had a sad loss of his girlfriend, …a bit of alcohol abuse … and he had to have chemo/radiation for anal cancer. So it was pretty tricky to get him to comply…. that's when the liaison officer really came to the fore”* Hospital Cancer Coordinator 5 (Non-Indigenous). “*So the family came down as escorts and guardians and it wasn't just the mother and father, it was siblings as well, so they couldn't just leave them with anyone….we'd have to make sure that we'd call ….and that they were OK up there”*. Hospital ALO 8.
Determining patient needs	“*So in our introduction it's always that we let them (patient) know that they direct us as to what they need, so needs and concerns, that's what we're there for”* Hospital ALO 8. “*The discussion around what their (Indigenous patients) cancer might be and the treatment that would entail would be very similar but we do know that we have more social issues with Aboriginal patients and so we do get more social work involvement and more support around them and we work with the liaison officer to come in as well”*. Non-Indigenous Hospital Radiation Oncologist 11.
Provision of practical assistance	“*I've got a lady who comes down and every time she asks me to get her an earlier (appointment)…she comes from x [4 hours away from Brisbane], so she leaves so early in the morning and she doesn't get home until late at night”*. Hospital ALO 7 “*So, it's assistance with food, accommodation, transport; they're the main big ones. Then it's just emotional and cultural support”*. Hospital ALO 8
Advocates for Indigenous Health	*I had a patient …. covered in cancer and they (hospital doctors) wanted him to sign a not for resuscitation which goes against everything (the patient) believes in and refused palliative care because he didn't want to give up. It was really hard trying to get the treating team to get that*. Hospital ALO 7 *I feel very strongly that part of the role of any practitioner (particularly in Indigenous health setting) is that advocacy and liaison for their client no matter what the situation or need is… there's no structured format for that. It's very much on what is required per person*. PHC GP 15 (non-Indigenous).

### Culturally Competent and Responsive Care

The majority of PHC setting participants clearly articulated that Indigenous patients were more likely to access cancer follow-up care if care was delivered in a culturally competent and responsive manner. Indigenous patients at times waited to see Indigenous staff members for their health checks and were more willing to discuss medical concerns with health staff of the same ethnicity. PHC interviewees spoke of how Indigenous patients often did not have a full understanding of their cancer treatment, and often sought clarity with Indigenous staff members rather than non-Indigenous staff members.

Indigenous health professionals showed recognition that spirituality, well-being and culture are intrinsically interwoven and central to the Indigenous perception of health and well-being. Some participants suggested that Indigenous health staff are better placed to understand and relate to this holistic view of health, and thus able to deliver culturally responsive care, with both aspects being seen as significant enablers to accessing cancer care. As one respondent shared,

“*…being Indigenous, it's a lot different to being non-Indigenous, because you know; you've got your spirituality and everything is not in the box and half the time what's outside the box is missed…”* PHC Indigenous Nurse 8.

Some interviewees spoke of health professionals working together as a team (regardless of Indigenous status) to provide culturally competent and responsive care and identified this as an important aspect of quality care for providing continuity of care for Indigenous patients. The provision of culturally responsive care that acknowledged and incorporated the importance of patients' spiritual beliefs was important. Culturally competent and responsive care as described by participants was more evident in the PHC setting and at the interface between the PHC and hospital setting, compared to during patient hospitalization.

### Psychological Support for Patients and Families

According to participants, the provision of psychological support for Indigenous cancer patients takes place in many forms including the visual presence of Indigenous health professionals, availability of Indigenous staff to respond to patients' needs and/or “having a yarn” to see how they are. Taking the initiative to seek-out and follow-up patients, providing support for the patient and family, accompanying patients to appointments, and helping to navigate the health system are also ways health staff provided psychological support.

“*What does it (emotional and cultural support) look like? I think it's, um, I think it's just presence. I think them knowing that there's a service here that can provide assistance … it's you know, it's the cultural support is there and emotional support, I think that's included with the presence. It's how we build the rapport”*. Hospital Aboriginal Liaison Officer 8.

From the perspectives of hospital health professionals, psychological support was at times delivered by more than one staff member, with the overlapping of some professional roles. Patient needs, referrals from hospital staff, availability of Aboriginal liaison officers (ALOs), and length of hospital stay were some of the reported factors seen as determining whether hospital ALOs provided psychological support to Indigenous cancer patients. However, based on interviews with hospital health staff, there appears to be some level of ambiguity regarding the role of the ALO and a lack of clarity of referral processes. For example, some health professionals reported automatic referral of Indigenous patients to the ALO upon hospital admission, whereas others described referrals on an “as needed” basis. In comparison, in the PHC setting, psychological support was reported to be offered to Indigenous patients as a standard practice and was delivered in a consistent manner. PHC referrals were made to psychologists or other speciality staff in the PHC, secondary or hospital setting as required.

### Meeting Patient Needs

Interviewees perceived that Indigenous cancer patients appeared to be more confident approaching Indigenous health professionals and those they had previously established rapport with (regardless of Indigenous status) to discuss their cancer condition and general health issues. This was especially evident in the PHC setting. It was reported that Indigenous patients sometimes felt overwhelmed, confused and vulnerable accessing cancer-related services and treatment. Some patients experienced uncertainty regarding the impact of a cancer diagnosis on their family, and how to proceed with treatment. Family responsibilities and socio-economic concerns were identified as other challenges for some patients when attempting to access cancer treatment.

Respondents observed that Indigenous health professionals play a crucial role in understanding Indigenous patients' concerns and “talking through” these concerns with patients, determining patient needs and how best to provide meaningful assistance, providing psychological support, and patient education in a culturally safe manner.

“*There's no rule or policy. No person is the same, so, I ask first, I ask the patient what they would like me to do, what do they need from me? Do they need transport, support, family support…and then they can say that they don't need me or they need me”*. PHC Indigenous Care Coordinator 2.

Interviewees across both PHC and hospital settings expressed genuine interest in determining and understanding Indigenous patients' needs. However, there appeared to be more challenges in the hospital setting, relating to heavy workloads for staff and time pressures (for example, two ALOs for the ~800 bed hospital), unclear ALO referral pathways, uncertainty regarding scope and role of the ALOs, and cultural barriers. Furthermore, difficulties determining the Indigenous status of cancer patients attending the hospital were reported to be a challenge for some hospital staff. In contrast, participating PHC services had a higher number of Indigenous staff employed, and issues relating to determining Indigenous patients' identity were not raised by interviewees from ACCHSs. Based on interviewees' feedback, staff of PHC services were more confident than hospital staff in being able to meet the needs of Indigenous cancer patients.

### Practical Assistance

Most participants spoke about the additional challenges Indigenous cancer patients experience accessing cancer treatment and services especially in the hospital setting. The main barriers identified in this study relate to finances, transport, and accommodation (especially when travel for treatment is required). Other concerns identified by the participants included the patient's own caring responsibilities, and the flexibility of appointment times to fit in with available transportation schedules. According to some interviewees, Indigenous health professionals were placed in a better position to provide practical assistance and respond to identified patient needs.

“*…a lot of our people don't like asking for help… especially if it's non-Indigenous. It's a pride thing as well as other stuff. A lot of the time they don't even ask”*. PHC Indigenous Nurse 14.

All PHC centers in this study were able to provide reasonable levels of practical assistance such as transportation services for Indigenous patients to attend PHC and hospital appointments. In comparison, although the hospital provided some practical assistance, according to some participants, this did not appear to be as well-coordinated.

### Advocates for Indigenous Health

Participants spoke about the importance of Indigenous health staff members as advocates for Indigenous patients in accessing cancer care and supporting Indigenous cancer patients' needs especially in the PHC setting and at the interface between the health sectors. Examples of such advocacy roles included: organization, follow-up of patient appointments, electing to be the patient's contact person, and taking on the “trouble shooter” role to manage unforeseen difficulties. Advocacy roles also extended to ensuring patients received continuity of care and quality care at the PHC and hospital interface level and, where possible, ensuring patients did not slip through the gaps in the health care system.

Other roles included locating, accompanying and transporting patients to appointments to encourage attendance, as well as, taking on the role of interpreter to explain cancer treatment pathways in ways patients could understand. One Indigenous health professional respondent conveyed that she spoke on behalf of her patients as needed. Where necessary, Indigenous health staff advocated for improvements to services to benefit patients as shown in the following statement.

“*In the past we only had two days a week (transport service) going to [regional city] for specialist appointments and I didn't agree with that myself as a health worker… the specialists come certain days and that's it, you've got to get the patients up there, and that's when I said that I'd fight for my community to make sure that it was every day”*. PHC Indigenous Health Worker 17.

## Discussion

Participants reported that Indigenous health professionals were found to contribute significantly across all themes in cancer follow-up care for Indigenous patients in the PHC setting, and at the interface between the PHC and tertiary setting. Culturally competent and responsive care, psychological support, determining patient needs, provision of practical assistance, and advocating for Indigenous health were identified as being key aspects in follow-up cancer care in this study. Central to, and underpinning these identified themes is the delivery of culturally competent care.

Indigenous health professionals play an important role in the provision of culturally responsive care. One way to understand this significance is in relation to the historical context of Indigenous peoples' experiences in Australia. This includes the consequences of colonialism, forced removals from family and other past government discriminatory policies, experiences of racism (23), and previous negative experiences accessing the health system. Thus, trust, rapport, and cultural safety are crucial when Indigenous patients attempt to navigate the mainstream healthcare system.

Indigenous health staff appeared to sustain better connection, rapport, and trust with Indigenous patients as reported from our study sample. The main reason for this could be that Indigenous professionals already possess a level of baseline knowledge of Indigenous culture, and thus bring with them the cultural competency and understanding of how deeply culture is embedded into the health and well-being of Indigenous people. This is supported by other research showing that people in general prefer to engage with health professionals from the same ethnic background as themselves, and that this improves health outcomes (24, 25).

Some participants in our study reported Indigenous cancer patients' reluctance in accessing cancer services, such as not reporting cancer symptoms, reluctance to attend specialist cancer appointments at hospital, and fear of seeking clarification from a hospital specialist about their cancer condition. In this study, such barriers were counteracted by the support and assistance of Indigenous health professionals. Participants also reported and acknowledged the importance of all health professionals working together to provide quality culturally competent and responsive care.

The availability of well-designed cultural safety programs for non-Indigenous professionals to improve knowledge of Indigenous culture could assist non-Indigenous and Indigenous health staff work together more effectively in teams and could also improve the delivery of culturally responsive health care (26). As reflected in the Aboriginal Torres Strait Islander Health Performance Framework, a health workforce that is culturally and clinically appropriate will provide a service that is responsive to needs, and committed to the health outcomes of Indigenous patients (2).

One study that examined issues relating to the cultural responsiveness of PHC services for Indigenous people in a remote community in Queensland found a significant difference in perceptions between the staff and Indigenous patients in providing culturally appropriate healthcare. Non-Indigenous PHC staff perceived that communication was clear and understandable; the provision of culturally appropriate services was satisfactory and a good level of support was provided. However, Indigenous patients accessing the health service did not share these perceptions (27). The presence of Indigenous health staff in a service helps build the competency of the non-Indigenous workforce, and actively talking about cultural diversity can potentially result in Aboriginal cancer care being more culturally inclusive (28, 29).

Participants from ACCHSs in this study reported more opportunity and were more confident in meeting the needs of Indigenous cancer patients compared to those at the hospital service. This could relate to a number of reasons including: in the PHC service setting, health services appeared to have processes in place to support stronger teamwork amongst staff within the organization; Indigenous health services are generally smaller organizations compared to a tertiary level hospital and solely focus on improving Indigenous health outcomes. These services are also supported by strong organizational policies supportive of a holistic view of health and a commitment toward improving Indigenous health outcomes. Other reasons for providing more opportunity to attend to Indigenous patients' cancer needs include employing a high proportion of Indigenous staff resulting in reducing cultural barriers, more opportunity to establish and maintain long-term relationships with patients given services are within the community setting, and less competition for scarce resources at the organizational level compared to a tertiary hospital service.

A recent Queensland-based study also found that Indigenous people chose to travel more than 30 min, in some instances bypassing mainstream medical services in major cities, to access ACCHSs that were designed to focus on the needs of Indigenous people (30). The study also suggested that potential barriers around accessibility, acceptability, affordability and accommodation were to some degree overcome through support from ACCHSs (30), which appears to be consistent with the findings of this study. Furthermore, indicative of Indigenous support of ACCHSs since the increase in Commonwealth-funded Indigenous PHC organizations in Australia (from 1999–2000 to 2014–15), the number of episodes of care for these organizations have almost tripled (from 1.2 to 3.5 million) as has the number of full time staff equivalents in Australia (2).

### Implications for Health Services and Policy

Our findings provide insight into the contribution of Indigenous health professionals in the provision of culturally competent and responsive care. Indigenous health staff need to be adequately supported in their roles and appropriate training opportunities should be provided. However, given the Indigenous population represents only 3% of the total Australian population (3) and is significantly underrepresented in the health workforce, (2) additional strategies need to be implemented to provide culturally competent cancer care for the Indigenous population and to address the Indigenous health workforce shortage. One way to address this shortage is to implement strategies that promote better educational pathways to encourage Indigenous peoples' participation and completion of tertiary level courses within health disciplines. The provision of support, ongoing professional development/training opportunities, and appropriate staff retention incentives for existing staff could also be considered to encourage staff retention (31).

It is crucial for Indigenous and non-Indigenous staff to work in collaboration to deliver culturally responsive cancer services thus underscoring the importance of having effective policies and practices in place that support and promote collaborative teamwork (26). As participants from ACCHSs reported higher satisfaction levels compared to hospital staff in supporting the needs of Indigenous cancer patients, hospitals might consider how suitable components of ACCHSs' health care delivery models might be applied in hospital settings to enhance their delivery of culturally competent services for Indigenous cancer patients.

Appropriate systems and processes need to be refined and implemented to allow formal identification of patients' Indigenous status at the point of health service access. Challenges around the identification of Indigenous cancer patients are not unique to the hospital setting and are also evident in the PHC setting (13). Policies and guidelines that provide further clarity around existing referral pathways (32) and scope of practice of ALOs in the hospital setting could also be beneficial to minimize confusion and to prevent patients falling through the health care gaps.

### Limitations and Strengths

This study may not be representative of all Queensland Indigenous PHC services due to its small sample size, and lack of representation from government-operated and mainstream PHC services. The study sample included participants from one tertiary hospital. A study limitation is that it presents findings based solely on the viewpoints of health professionals on follow-up cancer care and does not capture the patients' perspective. However, patients' perspectives and voices on follow-up cancer care were explored in a separate but related study (8). Therefore, care may need to be exercised in generalizing from the study results. Nevertheless, this study provides valuable insights into how Indigenous health professionals contribute toward the provision of culturally competent cancer care in Queensland. Strategies undertaken to minimize study limitations included offering participants the opportunity to review their interview transcripts, two investigators independently analyzing the data, and drawing on the wider literature to interpret study results.

## Conclusions

Indigenous health professionals are placed in a unique position in increasing the accessibility of follow-up cancer care services and in supporting the needs of Indigenous cancer patients in Queensland. However, a collaborative effort is essential as non-Indigenous health staff also need to be sufficiently skilled to deliver culturally responsive follow-up care to Indigenous cancer patients.

## Author Contributions

PV, RB, and JA contributed to the design of the larger NHMRC study. ADW, PV, RB, and FC contributed to the design and methods of this study. ADW collected the data and conducted the data analysis. ADW interpreted the data with the support of FC and RB. ADW drafted the manuscript and all authors revised it critically, contributing to its editing, and approved its final version.

### Conflict of Interest Statement

The authors declare that the research was conducted in the absence of any commercial or financial relationships that could be construed as a potential conflict of interest.
